# COX Inhibition Profile and Molecular Docking Studies of Some 2-(Trimethoxyphenyl)-Thiazoles

**DOI:** 10.3390/molecules22091507

**Published:** 2017-09-09

**Authors:** Smaranda Dafina Oniga, Liliana Pacureanu, Cristina Ioana Stoica, Mariana Doina Palage, Alexandra Crăciun, Laurentiu Răzvan Rusu, Elena-Luminita Crisan, Cătălin Araniciu

**Affiliations:** 1 Faculty of Pharmacy, “Iuliu Hatieganu” University of Medicine and Pharmacy, 8 Victor Babes St, Cluj–Napoca 400012, Romania; smaranda.oniga@umfcluj.ro (S.D.O.); Stoica.Cristina@umfcluj.ro (C.I.S.); araniciu.catalin@umfcluj.ro (C.A.); 2 Institute of Chemistry Timisoara of Romanian Academy, 24 M. Viteazul Ave., Timisoara 300223, Romania; lumycrisan@yahoo.com; 3 Faculty of Medicine, “Iuliu Hatieganu” University of Medicine and Pharmacy, 8 Victor Babes St, Cluj–Napoca 400012, Romania; acraciun@umfcluj.ro (A.C.); imunorusu@yahoo.com (L.R.R.)

**Keywords:** 2-(trimethoxyphenyl)-thiazoles, selective COX-2 inhibition, NSAIDs, molecular docking

## Abstract

Non-steroidal anti-inflammatory drugs (NSAIDs) are commonly used therapeutic agents that exhibit frequent and sometimes severe adverse effects, including gastrointestinal ulcerations and cardiovascular disorders. In an effort to obtain safer NSAIDs, we assessed the direct cyclooxygenase (COX) inhibition activity and we investigated the potential COX binding mode of some previously reported 2-(trimethoxyphenyl)-thiazoles. The in vitro COX inhibition assays were performed against ovine COX-1 and human recombinant COX-2. Molecular docking studies were performed to explain the possible interactions between the inhibitors and both COX isoforms binding pockets. Four of the tested compounds proved to be good inhibitors of both COX isoforms, but only compound **A3** showed a good COX-2 selectivity index, similar to meloxicam. The plausible binding mode of compound **A3** revealed hydrogen bond interactions with binding site key residues including Arg120, Tyr355, Ser530, Met522 and Trp387, whereas hydrophobic contacts were detected with Leu352, Val349, Leu359, Phe518, Gly526, and Ala527. Computationally predicted pharmacokinetic profile revealed **A3** as lead candidate. The present data prove that the investigated compounds inhibit COX and thus confirm the previously reported in vivo anti-inflammatory screening results suggesting that **A3** is a suitable candidate for further development as a NSAID.

## 1. Introduction 

Non-steroidal anti-inflammatory drugs (NSAIDs) that act by cyclooxygenase inhibition are a major drug class. Due to their ample therapeutic use that ranges from the treatment of fever and mild pain up to severe chronic inflammatory disorders, NSAIDs are one of the most commonly used medicines. The wide scale, frequent and sometimes long-term use of these drugs has allowed for a very good characterization of their safety profile. While some adverse reactions concerning gastrointestinal manifestations (gastritis, ulcer, bleeding) are well documented and established, others, like the cardiovascular risk, are still being assessed today [1,2]. 

It is well established that NSAIDs act by blocking the production of pro-inflammatory prostaglandins through the inhibition of cyclooxygenase (COX). At least two isoforms of COX are known, COX-1 and COX-2. COX-1 is mainly considered a “housekeeping enzyme”. It is widely distributed in most tissues where it performs mainly physiological roles like: protecting the gastric mucosa, kidney function maintenance and protection, or regulating platelet aggregation via stimulating thromboxane A2 (TXA2). By contrast, COX-2 is viewed primarily as responsible for the initiation and maintenance of the inflammation process with only minor physiological roles like stimulating prostacyclin (PGI2) production and thus preventing platelet aggregation [3,4,5]. 

It is commonly accepted that gastrointestinal side-effects are mainly associated with the inhibition of the cyclooxygenase-1 (COX-1), while cardiovascular side-effects are directly linked with the inhibition of COX-2 (possibly by blocking PGI2 biosynthesis while not hindering TXA2 formation [4]). The withdrawal from the market of most COX-2 specific inhibitors, coxibs (valdecoxib, rofecoxib), has proven that the intent to decrease gastrointestinal side-effects by creating specific COX-2 inhibitors has turned out to be unfavorable, as they are characterized by significantly higher cardiovascular risks [6]. It seems that the higher the specificity towards COX-2 inhibition, the higher the risk of CV undesirable effects. This observation is supported by the fact that celecoxib, the only coxib that is still approved by the US Food and Drug Administration (FDA), is actually the least COX-2 specific of all coxibs and thus shows a higher percentage of COX-1 inhibition than other coxibs [7].

Considering this, we aimed to obtain molecules that act only as selective COX-2 inhibitors, but are not specific COX-2 inhibitors and still maintain some degree of COX-1 inhibition. To this effect, we set out to mimic the pharmacological profile of meloxicam, which is only slightly COX-2 selective and not COX-2 specific. Optimally, the new derivatives should have a COX-1/COX-2 selectivity ratio higher than meloxicam but lower than celecoxib [7].

**Figure 1:**
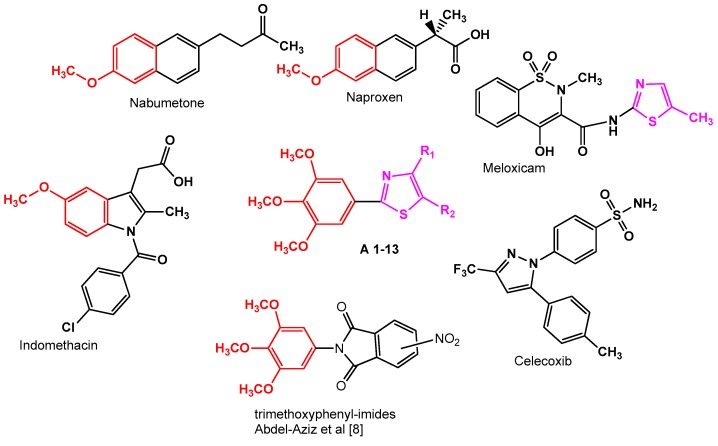
The inspiration for the design of the 2-(trimethoxyphenyl)-4-R1-5R2-thiazole scaffold.

**Figure 2:**
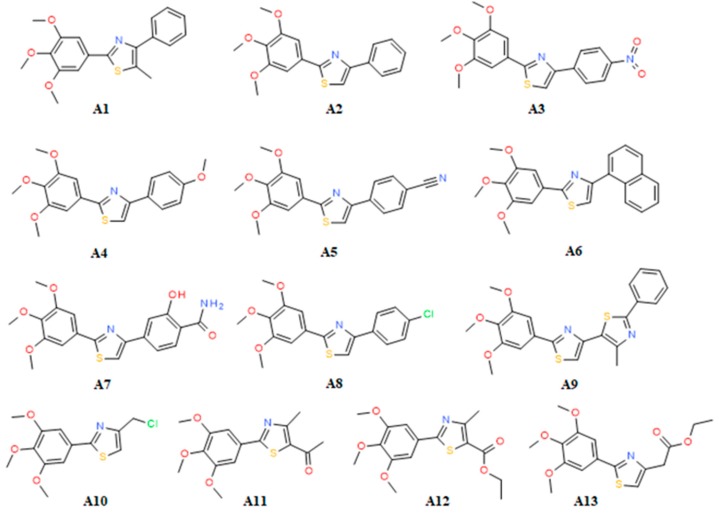
Chemical structures of the compounds **A1**–**13**.

Thus, inspired by the classical NSAIDs, molecules which contain methoxy groups (nabumetone, indomethacin, naproxen) and also recent research undertaken by Abdel–Aziz et al. [8], as shown in Figure 1, we previously designed and synthesized a series of 4,5-substituted 2-(trimethoxyphenyl) thiazoles [9] (**A1**–**13**, Figure 2). The thiazole nucleus is a well established component of many drugs [10,11,12] and was chosen as a key moiety because it is present in known NSAIDs (meloxicam, fentiazac) and also in many lead anti-inflammatory molecules that are under development: 2-aryl-thiazole [13], furo[2,3-d]thiazole [14], coumarin-thiazoles [15], diarylthiazoles [16].

In order to assess the importance of the diaryl-heterocyclic structures of coxibs on selective COX-2 inhibition, we previously designed and tested molecules with two, three or even four (hetero)aromatic rings [9,17,18]. In our previous papers, we also described the preliminary evaluation of the anti-inflammatory potential of the compounds by determining their effects using an induced acute inflammation experimental model [9,18].

Encouraging results obtained in our previous study have prompted the necessity of determining the direct COX-1/2 inhibitory potential of compounds **A1**–**13**, as well as the evaluation of the selectivity ratio. This was performed by using in vitro COX inhibitor screening assays. Furthermore, molecular docking studies were performed in order to predict the binding mode of compounds **A1**–**13**, based on our previous expertise in this field [19,20].

## 2. Results and Discussion 

### 2.1. In Vitro Cyclooxygenase Inhibition Assay

The COX-1/2 inhibitory activities of the tested compounds were evaluated using the enzyme immunoassay (EIA) method against ovine COX-1 and human recombinant COX-2. The half maximal inhibitory concentrations IC_50_ values calculated from experimental data are shown in Table 1. The selectivity index was calculated as the ratio IC_50_ COX-1/IC_50_ COX-2. The results obtained for the standard NSAIDs are similar to those described by the kit manufacturer. The tested compounds showed variable array of COX-1/2 inhibition potential. While most of the tested compounds inhibit COX-1 and COX-2, only a few of them have an inhibitory activity of IC_50_ < 100 μM that is comparable to meloxicam and other NSAIDs.

Half maximal inhibitory concentration for COX-1 is achieved by concentrations of 26.88 μM in the case of compound **A6**. Compounds **A2**, **A4**, **A7**, **A8** also have a potent COX-1 inhibitory effect. Half inhibitory concentration for COX-2 is achieved by concentrations as low as 23.26 μM in the case of compound **A2**. Compounds **A3**, **A6**, **A8** also have a potent COX-2 inhibitory effect with IC_50_ < 30 μM.

When considering the selectivity index as well as the inhibitory potency, the compounds **A2**, **A6** and **A8** prove to be active at low doses but have little selectivity towards COX-2, as they have similar activity against both enzyme subtypes.

The most promising inhibitor seems to be compound **A3**, which has a significant degree of selectivity towards COX-2, without being specific to it. From this perspective, compound **A3** seems to be very similar to the standard meloxicam. These findings are in agreement with the results obtained by other researchers [8] suggesting that a trimethoxyphenyl moiety together with NO_2_ substituent on the phenyl cycle leads to a good anti-inflammatory effect.

When we compare the in vitro results with our in vivo findings from previous paper [9], the result sets are consistent and support one another. Compounds **A3** and **A8** proved to be efficient in vivo anti-inflammatory agents, and, considering the current data, we can now state that they act by COX inhibition. By contrast, compound **A2** showed a good COX inhibitory effect in vitro, but lacked the in vivo effect [9]. This is most likely due to off-target effects or deficiencies in the pharmacokinetic profile that prevented the compound to reach the target enzyme in vivo. Regarding compounds **A4**, **A5**, **A6**, **A7**, **A8**, the existence of anti-inflammatory potential was confirmed, but, as already suggested by the in vivo research, the doses required are higher than those of the standard meloxicam [9].

### 2.2. SAR

The experimental results showed that four of the synthesized compounds (**A2**, **A3**, **A6** and **A8**) were more active than the rest of compounds, having IC_50_ values (23.26 μM to 28.87 μM) about two times higher than clinically used meloxicam (12.50 μM), but only compound **A3** showed selectivity for COX-2 (Selectivity Index SI = 9.24), similar to meloxicam (SI = 11.03). Compound **A2** was the most potent inhibitor in this series with the COX-2 inhibitory activity (IC_50_) of 23.26 μM. Compounds **A2**, **A6** and **A8** also displayed low half-maximal inhibitory concentrations for COX-1 (IC_50_ = 26.88–34.53 μM). Compound **A9**, which contains an extra 1,3-thiazole ring with respect to the rest of compounds, displayed lower potency for both COX-1 and COX-2 (83.16 μM, and 70.86 μM, respectively), lacking also isoform selectivity (SI 1.17). However, the presence of a 4-methyl substituent on the second 1,3-thiazole ring may exhibit a significant influence. For instance, when R_2_ is a methyl group (5 position on thiazole core), the compound is practically inactive against COX-2 (compound **A1**) in comparison with its non-methylated counterpart (compound **A2**). A methyl substituent probably induces a steric clash with COX-2 distinctive pocket residues. Compound **A7** displayed low selectivity for COX-1 (SI = 0.51) and weak potency for COX-2 (IC_50_ 105.67 μM), see Table 1. 

The presence of substituents at the 4 position of the phenyl ring (compounds **A3**, **A4**, **A5**, **A7**, **A8**) does not lead to the increase of affinity with respect to the unsubstituted derivative (compound **A2**), but the resulting selectivity for COX-2 is variable. Thus, the –NO_2_ group provided significant selectivity for COX-2, whereas –O–CH_3_ (compound **A4**) and –CN (compound **A5**) decreased the affinity for both isozymes and the selectivity for COX-2 (see Table 1). More interestingly, when the phenyl substituent is replaced with naphthyl, as in compound **A6**, the affinity was preserved, but selectivity was lost (SI=0.93). The naphtyl substituent probably allows similar hydrophobic interactions with both isozymes. The 3 –OH and 4 –CO–NH_2_ moieties (compound **A7**) decreased the affinities for both isozymes showing a stronger effect on COX-2, whereas 4 –Cl (compound **A8**) preserved the affinity and caused a slight preference for COX-2. The remaining compounds displayed only weak biological activity and practically can be considered inactive (IC_50_ > 70μM). The effects of the substituents of the thiazole ring on potency and selectivity (compounds **A3** to **A13**) were different and dependent on the nature of the R_1_ and R_2_ substituents, respectively aromatic or aliphatic. The substitution with –CH_3_, –CH_2_Cl, –CH_2_–COO–C_2_H_5_ at R1 and –COCH_3_, –COOC_2_H_5_ at R_2_, induced the loss of biological effect (compounds **A10**, **A11**, **A12** and **A13**). Thus, we can observe that the introduction of an aromatic substituent at position R_1_ led to increased potency towards both COX isoenzymes, but the presence of aliphatic electron donating or withdrawing groups decreased the COX inhibition potency. The lessening of affinities of compounds **A10**–**13** might be explained on the basis of the non-aromatic nature of the substituents. According to previous investigations, the aromatic core is very important for the affinity [21]. Hydrophobic substituents, such as naphtyl (compound **A6**), preserve the potency with respect to compound **A2**, but shift the selectivity towards COX-1, whereas Cl (hydrophobic plus hydrogen bonding) showed a slight preference towards COX-2. The nature of the substituent at the 4 position of the phenyl ring proved to be directional, being dependent on the electronic properties, i.e., the introduction of a nitro group (compound **A3**) increased COX-2 selectivity, the resulting effect was a selective inhibition of COX-2 (SI = 9.242).

### 2.3. Docking

Structural differences among the binding sites of COX-1 and COX-2 provided valuable guidelines for the design of selective COX-2 inhibitors [22,23,24]. The main difference consists in the existence of a second pocket inside of COX binding site, which is more accessible in COX-2 because of the replacement of Ile523 in COX-1 with a smaller side chain residue Val523, linked with conformational changes at Tyr355, which opens up the hydrophobic chain of the additional pocket including Leu352, Ser353, Tyr355, Phe518 and Val523 [23]. In COX-1, this pocket is not accessible due to the larger volume of Ile523. The access to this additional pocket is promoted by a further isoleucine to valine substitution at position 434, whose side chain packs against Phe518 creating a molecular doorway that opens to the second hydrophilic pocket [23]. On the contrary, in COX-1, this door is closed due to the larger side chain of isoleucine. In this manner, the amino acid at position 434 contributes significantly to the selectivity. Another structural difference is registered at position 513 where histidine is substituted by arginine in COX-2 [25]. However, COX-1/2 displays almost identical catalytic sites; nevertheless, the sequence homology is merely 65% [22]. Hence, in the case of meloxicam, a slightly different binding pattern in COX-1 and COX-2 was observed [22].

#### 2.3.1. Validation of the Docking Protocol

**Figure 3:**
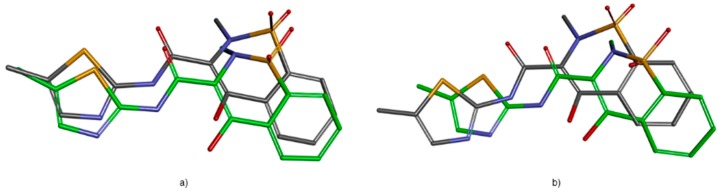
Overlay of meloxicam (MXM) conformation extracted from 4M11 (**a**) and 4O1Z (**b**) co-crystals (carbon shown in green) with the best docked conformer in cyclooxygenase (COX-2) and (COX-1) (carbon depicted in grey).

The identification of an appropriate docking protocol is a key step in the obtaining of reliable docking poses. To validate the docking protocol, meloxicam was docked into the crystal structures of COX-1/2 (PDB ID: 4M11, 4O1Z). Since meloxicam binds to COX-1/2 using two water molecules situated on each side of the ligand [22], the waters 25 and 117 in COX-1 and waters 84 and 161 in COX-2 were retained in order to obtain an unequivocal pose with respect to co-crystal configuration. The Induced Fit Docking (IFD) protocol reproduced well the interaction conformation of meloxicam with root mean squared deviation (RMSD) values of 1.407 Å (COX-2) and 1.475 Å (COX-1) (see Figure 3 and Table 2). Excepting a direct hydrogen bond interaction between 4-hydroxy moiety of benzothiazine and SER530, the meloxicam does not interact directly with binding site amino acid residues (Table 2) [22]. Particularly, meloxicam makes two hydrogen bonding networks with two highly coordinated water molecules to Tyr385/Ser530 (water 25 (COX-2)/water 117 (COX-1)) and Arg120/Tyr355 (water 84 (COX-2)/water 161 (COX-1)) [22].

Exquisite changes nearby Phe518, due to the replacement of Ile434 with valine in COX-2, result in different conformers of Phe518 in COX-1 and COX-2 and thus account for the selectivity of meloxicam for COX-2 [22]. On the contrary, rofecoxib and celecoxib take advantage of the substitution of Ile to Val at position 523 in COX-2 [23]. As can be observed the distances obtained by docking are close to those measured experimentally, which validate the accuracy of our docking protocol (Table 2). The direct interactions of meloxicam with Ser530 and water molecules, and the interactions between ligand-bounded waters and Arg120, Tyr355, Tyr385 and Ser530 were also reproduced.

#### 2.3.2. Mode of Binding

**Figure 4:**
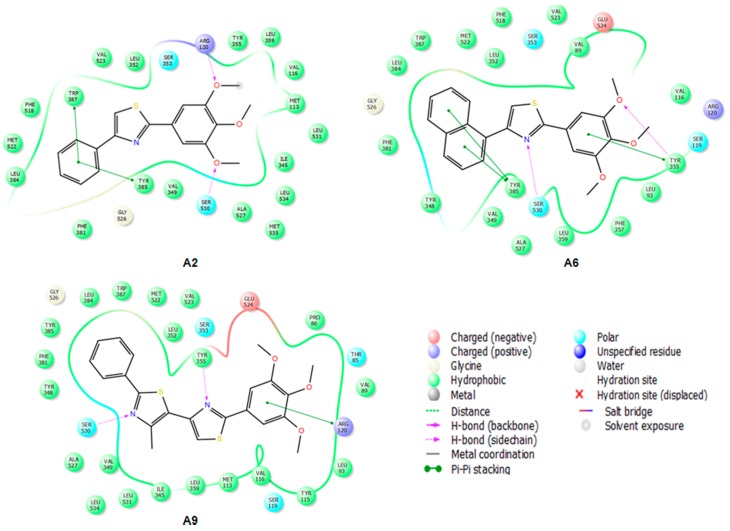
2D projection of the interactions of compounds **A2**, **A6** and **A9** with the active site of COX-2 (4M11).

**Figure 5:**
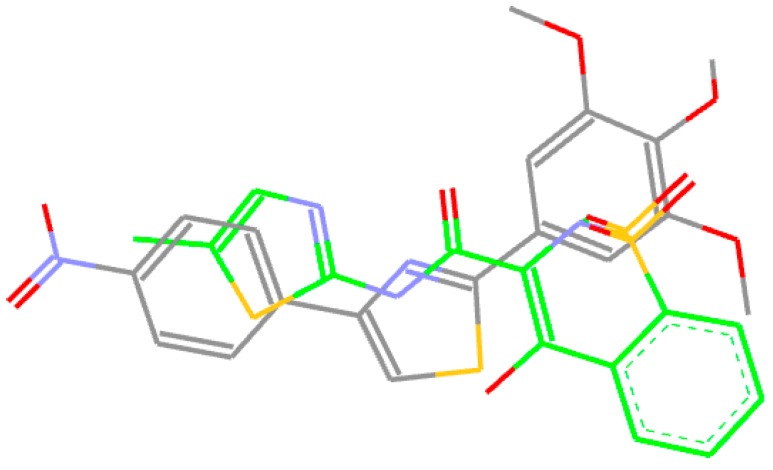
Overlay of MXM (carbon shown in green) and docked pose of **A3** (carbon depicted in grey).

**Figure 6:**
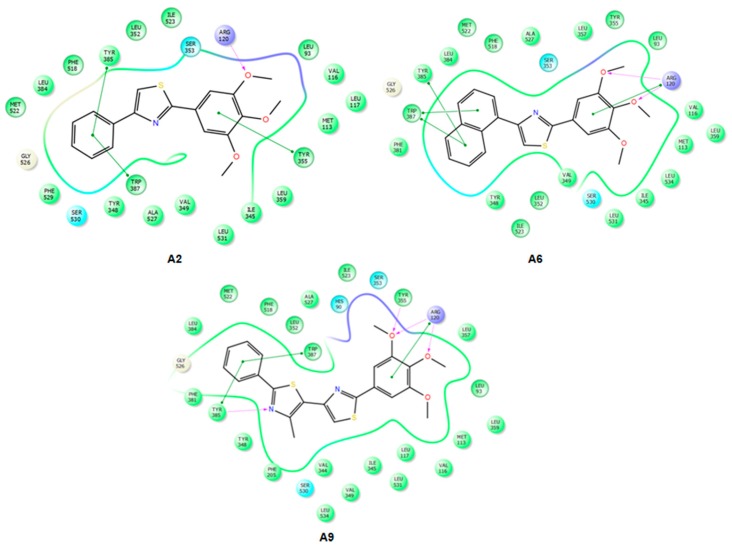
2D projections of the interactions of compounds **A2**, **A6** and **A9** with the active site of COX-1 (4O1Z).

**Figure 7:**
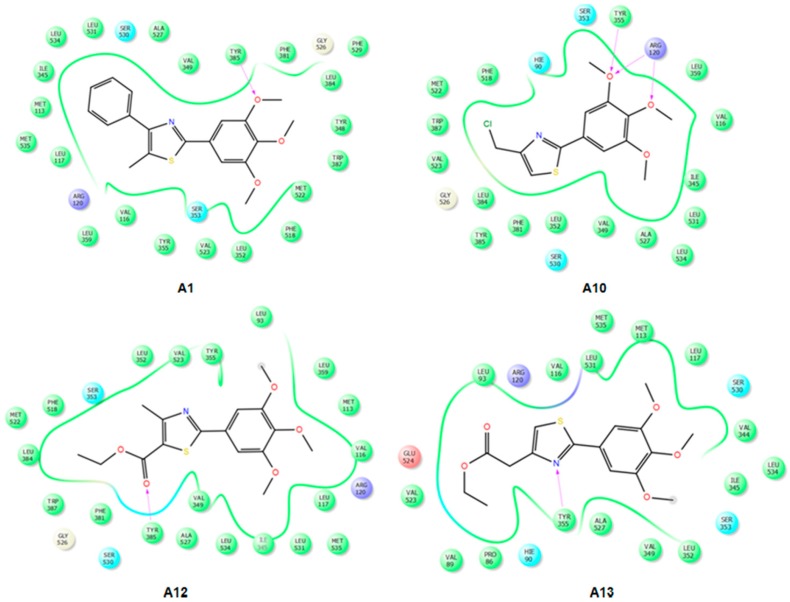
2D projections of the interactions of compounds **A1**, **A10**, **A12** and **A13** with the active site of COX-2 (4M11).

IFD docking outcomes include several induced fit configurations of the protein and implicitly a diversity of binding poses. The lowest energy poses of the most active compounds **A2**–**9** bind to the COX-1/2 hydrophobic channel with the trimethoxy-phenyl ring in close proximity of Arg120, aligned in a similar position with meloxicam (Figure 4 and Figure 5) having the substituents of the thiazole ring (R_1_, R_2_) directed towards the apex of the hydrophobic channel. We exemplified in Figure 4 the interactions observed for compounds **A2**, **A6** and **A9** into the COX-2 binding channel. As can be observed, these compounds occupy a close area as similar to that observed for meloxicam, where the following structural correspondences occur: (i) thiazine ring with 3,4,5-trimethoxyphenyl ring; (ii) carboxamide with thiazole; (iii) thiazole ring with 4-substituted phenyl ring (R_2_) (see Figure 5). Accurate hydrogen bonding interactions occur with crucial active site key amino acids Arg120 (OCH3 (**A2**)), Ser530 (*N*-thiazole (**A6**, **A9**)), and Tyr355 (*N*-thiazole (**A9**), trimethoxyphenyl (**A6**)), whereas hydrophobic interactions occur with Arg120 (trimethoxy-phenyl ring (**A9**)), Tyr355 (phenyl ring (**A2**) and naphtyl ring (**A6**)), Trp387 (phenyl ring (**A2**) and naphtyl ring (**A6**)), residues. Due to the high homology of binding site residues, similar binding interactions into COX-1 binding site (Figure 6) were observed for compounds **A2**, **A6**, and **A9**. In the case of the compounds **A1** and **A10**–**13**, we could not observe a predominant orientation and interaction pattern with binding pocket residues. In contrast, a larger number of distinct alternative poses were registered with respect to the compounds having an aromatic R_2_ substituent (Figure 7). Hence, compounds **A1**, **A10**, **A11**, **A12** and **A13** display lesser binding interactions with the COX-2 binding site. This could be caused either by the incompatibility between the narrow groove (Arg120 to Tyr355) and the bulkiness of the compound **A1** (R_2_ = CH3), or, in the case of less voluminous compounds **A10**, **A11**, **A12** and **A13**, by lesser interactions with binding site residues of COX-1/2 and an obvious inconsistency of their positions and interaction pattern with respect to the rest of compounds. However, in the case of compound **A1**, sterical hindrance within meloxicam binding domain in COX-2 may occur, i.e., methyl substituent of thiazole ring is situated in the close proximity of Leu359, Tyr355, Arg120, Leu117, Val116 residues, which are positioned in the immediate vicinity of distinct residues in COX-2 (Phe357, Lys358, His356, Tyr115, and Ser119) with regard to COX-1 (Leu357, Glu358, Phe356, Leu115, and Val119) (Figure 7).

#### 2.3.3. Docking of Compound **A3**

**Figure 8:**
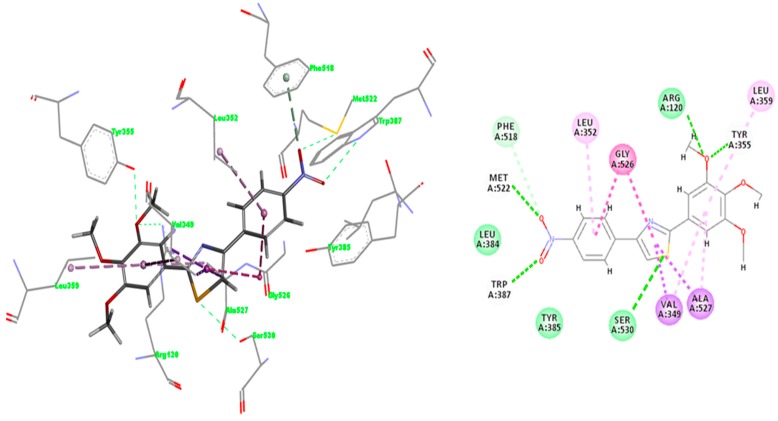
Induced Fit Docking (IFD) predicted binding mode of compound **A3** in COX-2 (PDBID: 4M11); H–bonds are depicted in green lines (Arg120 (3.070Å), Trp387 (2.922 Å), Met522 (2.811 Å) and Ser530 (3.000 Å), whereas hydrophobic interactions are shown in purple [28].

The affinity and selectivity of compound **A3** for COX-2 urged us to get insight into the plausible mode of interaction with COX-2 isozyme. The interaction pattern was selected according to the lowest energy poses of compound **A3** predicted by IFD score (Figure 7). The overlay of the best docked pose of compound **A3** and the meloxicam conformer extracted from 4M11 co-crystal is shown in Figure 5. Compound **A3** fitted well into COX-2 binding site occupying a similar region in the binding site as meloxicam, which allows the NO_2_ group to make hydrogen bonding interactions with Met522 and Trp387 at the apex of active site. Trp387 interact with bromophenyl ring of COX-2 selective celecoxib derivative SC-558 (PDBID: 1CX2) [23], whereas van der Waals contacts of cyclohexane group of NS-398 (PDBID: 3QMO), another COX-2 selective inhibitor, interacts with the side chain of Trp387 [26]. The NO_2_ group of compound **A3** engages a bifurcated H–bond with Trp387 and Met522 side chains. This pose might benefit from additional interaction energy due to the relative proximity of the Val116, which can generate an additive effect determining the selectivity for COX-2 [27]. The interaction forces require the NO_2_ moiety to embrace a particular orientation at the entrance into the hydrophobic channel. We can assume that these residues situated at the entrance into the side pocket of COX-2 are responsible for the preference of compound **A3** for COX-2, conferring stability to the complex [27]. The comparison of the interactions registered by meloxicam into 4M11 co-crystal and the docked pose of compound **A3** (Figure 8 [28]) shows that the S (thiazole) interacts with Ser530 by hydrogen bonding similar to benzothiazine 4-OH group of meloxicam.

Whereas compound **A3** interacts directly with Trp387 and Ser530, meloxicam interacts with Tyr385 and Ser530 by means of water 25. Tyr355 and Arg120 network with water 84 in 4M11 co-crystal, while compound **A3** interacts directly with these residues by the means of oxygen atom of methoxy group. However, a direct comparison with meloxicam binding mode can be plausible since structurally dissimilar ligands can occupy the same binding site of COX-2 (Figure 5) [26]. Compound **A3** (Figure 8) is inserted into a hydrophobic pocket rich in aromatic residues making hydrophobic interactions with Val349 (π-alkyl tri-methoxy-phenyl ring, π-σ thiazole ring), Leu352 (π-alkyl with nitro phenyl ring), Leu359 (trimethoxy-phenyl ring), Ala527 (π-σ thiazole ring), Phe518 (π-donor hydrogen bond nitro group), Gly526 (amide-π stacking nitro-phenyl ring), and Ala527 (π-alkyl tri-methoxy-phenyl ring). The 4-nitro-phenyl ring of **A3** is situated in the area of the active site surrounded by aromatic residues including Phe518, Tyr385 and Trp387. Site-directed mutagenesis pointed out that the preference of meloxicam for COX-2 arises due to slight changes nearby Phe518 generated by the substitution of Ile434 with Val [29]. Meloxicam undergoes a direct interaction mediated by 4′-methyl group with Phe518, in contrast to protein shift observed in the case of the celecoxib analogue SC-558 [23,29]. In this context, compound **A3** displays a binding pattern similar to that of moderately selective inhibitors of COX-2.

**Figure 9:**
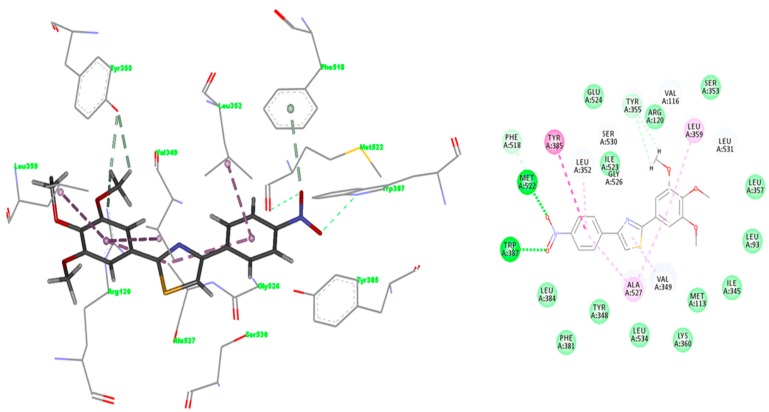
IFD predicted binding mode of compound A3 in COX-1 (PDBID: 4O1Z); H–bonds are depicted in green lines Trp387 (2.867 Å), Met522 (3.184 Å), whereas hydrophobic interactions are shown in purple [28].

The interactions registered by compound **A3** with COX-1 binding pocket (Figure 9 [28]) are similar to those registered in COX-2: two hydrogen bonds with Trp387 and Met522, whereas hydrophobic π-alkyl interactions appear with Leu352, Leu359, Val349, and Ala527 (two interactions). Carbon hydrogen bonds between the CH_3_ group belonging to the trimethoxy-phenyl ring occur with narrow groove residues Tyr355 and Arg120, and also one π-hydrogen bond with Phe518 was observed.

Hence, the exquisite differences in terms of binding site structural organization can be exploited, certifying our concept of medicinal chemistry design. Subtle structural differences registered by the hydrophobic channel can lead to significant particularities in terms of ligand selectivity [22]. The geometry of compound **A3** allows further insertion of substituents, with hydrogen bonding potential i.e., acceptor/donor groups to improve the affinity for COX-2. In the current work, we outline the identification of a selective COX-2 inhibitor, which will be subjected to further development by structural optimization.

### 2.4. Prediction of Pharmacokinetic Properties

Concerns regarding drug/lead likeness appear for compounds **A6**, **A8** and **A9** which break both the Rule Of Three (ROT) and Rule Of Five (ROF), and also compound **A5** trespasses only ROT (Table 3). However, drug-likeness (ROF) tolerates one rule breaking, but the compounds that fulfill thoroughly Jorgensen lead-like criteria are more likely to be orally available. Problematic human oral absorption appears in the case of compounds **A6** and **A9** (Table 3). Looking at Figure 2, one can observe the hydrophobic character of these compounds, respectively the absence of polar groups on phenyl/naphtyl ring with respect to the rest of compounds. Compound **A2**, which lacked a therapeutical effect in vivo, complies with ROF and ROT and shows good human oral bioavailability. In the case of our compounds, the increase of IC_50_ values for HERG K^+^ channels is necessary since values lower than −5 are not recommended. However, experimental determination and further multi-objective structural optimization (affinity, selectivity and pharmacokinetic properties) are needed to obtain more effective lead candidates with good bioavailability.

## 3. Materials and Methods 

### 3.1. In Vitro Cyclooxygenase Inhibitor Assay 

The cyclooxygenase inhibitory potential of compounds **A1**–**13** was assessed using the COX inhibitor Screening Assay Kit (Catalog No. 560131, Cayman Chemical, Ann Arbor, MI, USA). The kit utilizes an enzyme immunoassay (EIA) in order to quantify the prostanoid product resulted from COX catalyzed reaction. The inhibitory potential of our molecules was tested against the ovine COX-1 and human recombinant COX-2 enzymes. The COX inhibition reaction was performed by a 10 min incubation at 37 °C in the presence of reaction buffer, heme, COX-1 or COX-2 enzymes, and the tested inhibitor. The reaction was initiated by adding arachidonic acid and incubating at 37 °C for 2 min. Enzymatic catalysis was stopped by adding HCl. Stannous chloride was subsequently added to perform the reduction of COX-derived prostaglandin H2 (PGH2) produced in the reaction of COX leading to prostaglandin F2α (PGF2α). PGF2α was then quantified using the EIA kit. Prostanoid containing solutions obtained from the COX reaction were then diluted and transferred to plates that were pre-coated with monoclonal anti-rabbit IgG antibodies produced in mouse. They were incubated overnight in the presence of PG-acetylcholinesterase (AchE) conjugate and specific PG antiserum. The reaction mixture was then removed from the wells, the plates were washed to remove all unbound reagents and Ellman’s reagent (which contains the AchE substrate) was then added for plate development. After a 1 h incubation period, the yellow product of the AchE reaction was measured spectrophotometrically at 412 nm. The intensity of the color is inversely proportional to the amount of PG found in the sample. The PG quantity was determined using a PG standard curve generated on the same plate. All determinations were performed according to the manufacturer’s instructions and similar with other literature reports [30,31,32,33]. The IC_50_ values were calculated using a sigmoidal concentration-inhibition response curve (duplicate determinations). Every compound was assayed in the COX reaction on a range of concentrations from 0.03 μM to 300 µM in two distinct determinations. Each COX reaction sample was then EIA assayed at two dilutions and each dilution was tested in duplicate. Meloxicam, celecoxib and indomethacin provided by Cayman Chemical were used as reference compounds. Using IC_50_ values, the selectivity index (SI) was calculated as the ratio IC_50_COX-1/IC_50_COX-2.

### 3.2. Molecular Modeling

#### 3.2.1. Protein Preparation

Over the last few years, many X-ray crystal structures of COX-2 have been deposited in the Protein Data Bank (PDB) [25]. The structures of COX-1 and COX-2 co-crystalized with meloxicam were selected for docking experiments (PDBID: 4O1Z, 4M11) [22], and were processed in Maestro (Schrödinger, LLC, New York, NY, USA) [29] using the Protein Preparation Wizard facility [29]. The following preparation steps were completed: (i) protein structure integrity was checked and missing residues were added using Prime [29]; (ii) assign bond orders and add hydrogen atoms to the ligand molecule; (iii) add hydrogen atoms to protein heavy atoms and charge the Asp, Glu, Arg and Lys residues; (iv) optimize the orientation of hydroxyl groups on Ser, Thr and Tyr residues; (v) optimize the side chains of Gln and Asn residues; and (vi) determine the state of His residues. The ligand was retained throughout the protein preparation process. We prepared two versions of the receptor: with and without functional water molecules (waters 117/161 in COX-1, waters 25/84 in COX-2). The proteins, which include two functional water molecules, were used to dock meloxicam in order to validate the docking protocol, whereas the proteins without waters were designated for docking of compounds **A1**–**13**. The pocket was defined by selecting the ligand that is part of the meloxicam—COX-1/2 complexes. Finally, the COX-1/2- ligand complexes were assigned to geometry refinement using Optimized Potentials for Liquid Simulations (OPLS)–2005 force field restrained minimization, imposing an root mean square deviation (RMSD) of 0.3 Å for the convergence of heavy atoms. 

#### 3.2.2. Ligand Preparations

The ligands (meloxicam and compounds **A1**–**13**) were prepared using ligand preparation software LigPrep from Schrödinger suite [29]. Initially, the ligands were rendered in SMILES (simplified molecular-input line-entry system) strings, then a single low energy 3D conformer for each 2D structure, ionization states and tautomers in the pH range 7.4 ± 0.2 were generated, followed by optimization with OPLS 2005 force field, whereas charges were calculated using the MacroModel module implemented in the Schrödinger package using default settings [29]. The stereochemistry for the unassigned stereogenic centers was rendered, considering a maximum of 32 stereoisomers per ligand.

#### 3.2.3. Induced Fit Docking

Docking investigations performed under the assumption of a rigid receptor can produce inaccurate results because, upon ligand binding, proteins frequently experience side-chain or back-bone movements. The presence of a flexible area at the juncture of membrane binding domain with catalytic domain of cyclooxygenases allowing closed and open binding sites for NSAID [34] requires the use of flexible docking protocol. Therefore, we considered Induced–Fit Docking (IFD), which accounts for flexible binding domain of receptor, to predict the binding pattern of compounds **A1**–**13** with COX-1/2 [29,35,36]. IFD combines in an iterative fashion the ligand docking techniques with those for modeling receptor, to alter binding site conformation, which correspond to the most probable shape and binding motif of the ligand. IFD protocol rely on Glide [37] and Prime refinement algorithm [38], which provide precise docking results. The receptor grid was centered on the bound ligand (meloxicam). IFD includes the following stages: (i) constrained refinement of the protein within a RMSD of 0.18 Å; (ii) basic Glide docking of small-molecule allowing a softened potential keeping 20 poses/ligand, which display Coulomb-van der Waals score lower than 100 and hydrogen bond score lower than −0.05; (iii) protein–ligand complex was subjected to prime side chain conformational prediction for residues that display a distance of 5 Å to any ligand pose; (iv) the identical set of residues and ligand forming the poses are minimized with Prime, such as each pose geometry mirrors an induced fit; (v) accurate docking into induced fit receptor using Glide with default settings retaining the poses which fall under 30 kcal/mol with respect to the best pose (default); and (vi) calculation of the binding energy (IFD score) for any output pose [37,38]. Since meloxicam binds to COX-1/2 in a conformation that includes two water mediated networks, we conducted IFD docking of meloxicam in the presence of water molecules, whereas compounds **A1**–**13** were docked in the absence of water to avoid erroneous estimates of posses and binding energies. Ligand conformational sampling was carried out within a 2.5 kcal/mol energy window. The refinement was performed using the Prime molecular dynamics algorithm to account for binding domain flexibility within 5.0 Å of ligand poses [37]. The receptor and ligand softening potential was used with a scaling factor of 0.5 in both cases. Maximum 20 poses per ligand were saved. 

### 3.3. Prediction of Drug-Likeness and Pharmacokinetic Properties

The evaluation of “drug-likeness” and pharmacokinetic profile of thiazole derivatives was performed with the help of QikProp module implemented in Schrödinger package. QikProp was created by Professor William L. Jorgensen to estimate rapidly and accurately adsorption, distribution, metabolism, and excretion (ADME) properties [29]. Drug-likeness was assessed based on Lipinski’s “Rule of Five” (ROF) [39]. The “Jorgensen Rule-of-Three” (ROT) is based on the properties of more than 90% of 1700 oral drugs [40]. According to Jorgensen the thresholds for lead like properties include aqueous solubility logS > −5.7, – heterogeneous human epithelial colorectal adenocarcinoma (Caco-2) cell permeability should be higher than 22 nm/s, and less than seven primary metabolites [29]. QikProp can estimate meaningful physical descriptors and pharmaceutically significant properties for organic compounds.

## 4. Conclusions 

In conclusion, thiazole derivatives **A1**–**13** were evaluated as COX-1/2 inhibitors. Four of the tested compounds, **A2**, **A3**, **A6** and **A8** proved inhibitors of COX-1/2. Among them, compound **A3** exhibited potent COX-2 inhibitory activity and a selectivity index similar to meloxicam. Structure- activity relationship (SAR) investigation suggested that the substituents at position 4 of the phenyl ring –O–CH_3_, –Cl, –NO_2_, –CN and –CONH_2_ influenced markedly the selectivity for COX-2, although the affinity was not altered. The in vitro results give a firm indication regarding the mechanism of anti-inflammatory activity already proved by an in vivo study. For most compounds, a strong correlation between in vitro COX-inhibition and in vivo anti-inflammatory effect was established. 

Molecular docking studies suggested that the most active compounds **A2**–**9** can be positioned within the active sites of COX-1/2 similarly to meloxicam occupying the same subdomain, whereas weaker inhibitors (**A1**, **A10**–**13**) prefer another different orientation. The **A3**–COX-2 complex generated by docking, revealed intricate interactions with a COX-2 channel, including hydrogen bonds with key residues Arg120, Ser530, Met522 and Trp387 and hydrophobic interactions with Val349, Leu352, Leu359, Ala527, Phe518, Gly526, and Ala527. 

In the current work, we outlined the identification of a selective COX-2 inhibitor that will be subjected to further computationally assisted structural optimization to improve its potency and selectivity for COX-2.

## Figures and Tables

**Table 1:** Experimental IC_50_ (µM) against COX-1 and COX-2 for compounds **A1**–**13**.

IC_50_	COX-2	COX-1	SI	IC_50_	COX-2	COX-1	SI
C*	0.06	30.68	511.3	A6	28.87	26.88	0.93
M*	12.50	137.83	11.03	A7	105.67	54.72	0.51
I*	25.65	1.80	0.07	A8	25.64	31.46	1.22
A1	>300.00	140.10	0.007	A9	70.86	83.16	1.17
A2	23.26	34.53	1.48	A10	107.00	609.64	5.69
A3	25.50	235.67	9.24	A11	215.99	76.03	0.35
A4	75.00	50.37	0.67	A12	272.54	409.13	1.5
A5	148.40	73.26	0.49	A13	229.48	108.77	0.47

C*—Celecoxib, M*—Meloxicam, I*—Indomethacin; Cyclooxygenase (COX); The half maximal inhibitory concentration IC_50_—determined using sigmoidal concentration-inhibition curves; Selectivity index SI = IC_50_ COX-1/IC_50_ COX-2. The concentrations used for calculation of IC_50_ ranged from 0.03 μM to 300 µM.

**Table 2:** Hydrogen bond distances (Å) registered for meloxicam docked in cyclooxygenase COX-1 and COX-2 (Protein Data Bank (PDB) ID: 4M11 and 4O1Z).

H Bond	4M11	4O1Z
*N*-Thiazole .....HOH25/HOH117	2.853	3.044
HOH25/HOH117.....Tyr385	2.740	2.182
HOH25/HOH117.....Ser530	3.194	3.450
4-OH Benzothiazine.....Ser530	3.047	2.966
C=O Carboxamide.....HOH84/HOH161	3.184	3.638
HOH84/HOH161.....Tyr355	2.972	3.410
HOH84/HOH161.....Arg120	2.680	2.373
-NH Carboxamide..... 4-OH Benzothiazine	2.554	2.210
-C=O Carboxamide.....N Benzothiazine	2.613	2.711

**Table 3:** Pharmacokinetic properties calculated of compounds **A1**–**13**.

Molecule	QPlog HERG	QPP Caco	QPlogBB	QPP MDCK	QPlogKp	ROF	ROT	HOA
**A1**	−5.582	8697.865	0.408	7894.031	−0.473	0	0	3
**A2**	−5.698	8587.415	0.421	8547.499	−0.34	0	0	3
**A3**	−5.606	949.754	−0.653	790.214	−2.314	0	0	3
**A4**	−5.638	8587.208	0.359	8550.394	−0.435	0	0	3
**A5**	−5.741	1727.341	−0.381	1508.647	−1.726	0	1	3
**A6**	−6.158	8494.294	0.415	8471.98	−0.141	1	1	1
**A7**	−5.38	354.207	−1.157	272.109	−3.189	0	0	3
**A8**	−5.625	8586.23	0.595	10,000	−0.508	1	1	3
**A9**	−6.239	7022.555	0.382	9485.234	−0.47	1	1	1
**A10**	−4.516	8641.559	0.604	10,000	−0.992	0	0	3
**A11**	−4.406	2652.557	−0.163	2164.163	−2.012	0	0	3
**A12**	−4.706	1862.779	−0.445	1235.594	−2.23	0	0	3
**A13**	−5.088	1860.846	−0.478	1633.249	−2.022	0	0	3

QPlogHERG—predicted IC_50_ value for blockage of HERG K+ channels (human potassium voltage-gated channel); QPPCaco—predicted apparent heterogeneous human epithelial colorectal adenocarcinoma (Caco-2) cell permeability in nm/s; QPlogBB—predicted brain/blood partition coefficient; QPPMDCK—predicted apparent Madin-Darby canine kidney (MDCK) cell permeability in nm/s; QPlogKp—predicted skin permeability; ROF (Rule Of Five)—number of violations of Lipinski’s rule of five; ROT (Rule Of Three)—number of violations of Jorgensen’s rule of three; HOA (Human Oral Absorption)—predicted qualitative human oral absorption.
